# Removal of chromium (VI) from aqueous solution using vesicular basalt: A potential low cost wastewater treatment system

**DOI:** 10.1016/j.heliyon.2018.e00682

**Published:** 2018-07-10

**Authors:** Agegnehu Alemu, Brook Lemma, Nigus Gabbiye, Melisew Tadele Alula, Minyahl Teferi Desta

**Affiliations:** a Ethiopian Institute of Water Resources, Addis Ababa University, P.O Box 1176, Addis Ababa, Ethiopia; b College of Science, Bahir Dar University, P.O Box 79, Bahir Dar, Ethiopia; c College of Natural and Computational Science, Addis Ababa University, P.O Box 1176, Addis Ababa, Ethiopia; d Faculty of Chemical and Food Engineering, Bahir Dar University, P.O. Box 26, Bahir Dar, Ethiopia; e Botswana International University of Science and Technology, Private Bag 16, Botswana; f School of Earth Science, Bahir Dar University, P.O Box 79, Bahir Dar, Ethiopia

**Keywords:** Environmental science

## Abstract

In this study, vesicular basalt volcanic rock was taken and its application for adsorption of chromium (VI) from aqueous solution was investigated. Different physical and chemical properties of the powdered rock was studied using Fourier transform infrared spectroscopy (FT-IR), Powder X-ray diffraction (XRD) and scanning electron microscopy (SEM). A series of batch experiments were carried out to study the effect of various experimental parameters (pH, ionic strength and contact time) on chromium (VI) adsorption. It was found that the removal efficiency of chromium (VI) decreased with increasing pH and ionic strength. The adsorption process was optimal at pH 2. The maximum adsorption capacity was 79.20 mg kg^−1^ at an initial concentration of 5.0 mg L^−1^ and adsorbent dosage of 50 g L^−1^. In individual adsorption tests, Pseudo-second-order kinetic and Freundlich isotherm models could better describe chromium (VI) adsorption on the vesicular basalt. This study indicated that vesicular basalt, which is inexpensive, has the potential to remove chromium (VI) from polluted water.

## 1 Introduction

Contamination of water bodies with Cr (VI) occurs from natural and anthropogenic sources, the later with an ever-increasing impact on it. The main anthropogenic sources of Cr (VI) include metallurgy, refractory, electroplating, and production of chromium-containing compounds, such as pigments, paints, catalysts, chromic acid, and tanning agents [1].

Chromium occurs in the environment commonly as Cr (III) and Cr (VI) oxidation states, which have quite different chemical properties. Cr (VI) can be transformed to Cr (III) and vice versa depending on pH, the presence of oxidizing and reducing compounds, redox potential, the kinetics of the redox reactions, and the total chromium concentration in soil, water and atmospheric systems [2, 3, 4].

Cr (VI) is a powerful oxidant and many of its compounds are very soluble in water that makes it easily bioavailable [5]. Cr (VI) is the most toxic and its effects are carcinogenic [6, 7, 8]; mutagenic [9]; and teratogenic [10] to humans and animals. Other most sensitive noncancer effects of Cr (VI) compounds are severe respiratory (nasal and lung irritation), gastrointestinal (irritation, ulcer of the stomach and small intestine), haematological (microcytic, hypochromic anaemia), liver, kidney and reproductive organs damage and malfunctions such as decrease in sperm counts in males [11, 12]. According to WHO [13] drinking water guide line, the maximum permissible limit of Cr (VI) in potable water is 0.05 mg/L and USEPA [14] set a maximum limit of discharge 0.1 mg L^−1^ Cr (VI) to inland surface waters. Consequently, the removal of Cr (VI) from water and wastewater is very critical.

Different conventional methods are used for removal of Cr (VI), such as chemical precipitation [15], adsorption and filtration [16], ion exchange [17, 18]; Electrocoagulation [19], membrane separation [20] and electrodialysis [21]. However, these methods have some drawbacks such as low efficiency, high operating and maintenance cost, generate sludge causing disposal problems, or produce a secondary pollutant, which limits their applicability in the real situation [22].

In comparison adsorption method is simple to operate, solves the challenge of sludge disposal, and an effective method for the removal of Cr (VI) and other heavy metals from aqueous solutions. Though extra care and handling is required for the proper management of the used adsorbents and recovery of heavy metals. Some studies have revealed that adsorbents like activated carbon are effective in removing of a wide range of contaminants from water and wastewater, but they are not cost effective [23, 24, 25]. In view of its high cost there is continuing search for low cost potential adsorbents for the removal of Cr and other heavy metals from water and wastewater. An adsorbent is considered as a low cost if it requires little processing, abundant in nature and is a by-product or waste material resulting from an industry [26]. Several low cost adsorbents including clays [27, 28, 29, 30], industrial by-products [31, 32], agricultural wastes [33, 34], biomass [35], and polymeric materials [36], and young vesicular volcanic rocks [37] have been investigated for Cr (VI) removal.

Vesicular basalt rocks have unique properties as described below that deserve further investigation Vesicular basalt is a volcanic rock formed by rapid cooling of lava on the earth's surface. Pumice and scoria are the most abundant vesicular basalt rock types. Pumice is a white or grey finely porous rock frothy with air bubbles and rich in silica (felsic) whereas Scoria is texturally macrovesicular and more denser than pumice,silica-deficient (mafic) rock owning different colours ranging from red to black depending on its mineral composition [38, 39]. Vesicular basalt is abundant in many parts of the world such as Western Europe, Central America, Western South America, Western and northern part of the Pacific belt [40], Saudi Arabia [41], Central Africa [42] and East Africa [43]. Its abundance, variation in the chemical composition and variation of surface nature depending on the source composition and type of eruption of the magma received considerable interest to assess the ability of locally available vesicular basalt for the removal of Cr (VI) from wastewater.

Thus the objectives of this study were (i) to characterize the locally available vesicular basalt (VB), (ii) to assess the effects of pH, ionic strength, initial concentration of Cr (VI), and contact time on the adsorption of Cr (VI) in aqueous solutions, and (iii) to analyse the adsorption of Cr (VI) onto the VB surface using various kinetic and isotherm models. This experiment therefore attempts to shade some light to the understanding of capturing Cr (VI) is to give management sufficient time and ease of handling for safe disposal of this pollutant that happens to be released into the environment in countries like Ethiopia where control measures are inactive.

## 2 Materials and methods

### 2.1 Materials

The reagents potassium dichromate (≥99 % purity), diphenylcarbazide solution: prepared by dissolving 250 mg 1, 5-diphenylcarbazide (98 %) in 50 mL acetone (assay ≥99.5 %) following the standard procedures described in APHA [44]. All the reagents were obtained from Fisher scientific. Nitric acid (assay 68–70%) and potassium nitrate (99.0–100% assay) were purchased from BDH laboratory supplies. Sodium hydroxide pellets, (extra pure 98 %) was purchased from Research Lab Fine Chemical Industries and ultra pure water (conductivity = 0.05 μs cm^−1^), was obtained from Evoqua Water Technologies. All the reagents were of an analytical grade and used as received without additional purification. The grey VB rocks that were used in this research were collected around Bahir Dar City (North West of Ethiopia) close to Lake Tana and around the outlet of Abbay (Blue Nile) River. Its geographical location is at about 11°36′00″ N latitude and 37°24′00″ E longitude at an elevation of 1,800 m where all hills are mainly composed of this volcanic rock. The rocks were crushed using Geocrusher and sieved to decipher particle sizes ranging between 90 to 500 μm. These samples were washed with ultra-pure water and dried in an oven at 105 °C overnight. They were let to stand overnight to cool down to room temperature before they were used in the batch adsorption experiments then cooled to room temperature before use and made ready for the batch adsorption experiments.

### 2.2 Characterization of VB

Perkin Elmer Spectrum 65 Spectrometer (USA) was used to record the IR spectra in the mid infrared region (4000–400 cm^−1^) with a spectral resolution of 2 cm^−1^ using a pressed KBr pellet technique. The pellet was prepared by mixing approximately 1.0 % VB with 250 mg KBr and then finely pulverized and put into a pellet forming die. The minerals in the VB were identified by using a Bruker D2-phaser diffractometer using Cu Kα radiation of wavelength, λ = 1.54056 Å, with variable slits at 45 kV/40 mA. Scanning between 10 and 75 (2θ) at a scanning rate of 2° min^−1^ in steps of 0.02°. The morphological investigation and elemental identification of the VB was carried out using scanning electron microscope equipped with energy dispersive spectrometer (SEM-EDS), JEOL, JSM-6500F (Japan) at an accelerating voltage of 15 kV and a beam current of 1–3 nA.

### 2.3 Determination of pH of point zero charge (pHpzc)

The pHpzc of the VB samples was determined by batch equilibration technique [45]. 5 g of VB was added to a series of six flasks that contain each 100 mL of 0.01 mol L^−1^ KNO_3_ as a background electrolyte. The initial pH values were adjusted in the pH range of 2–11 using 0.1 mol L^−1^ of HNO_3_ or NaOH. Equilibration was carried out by shaking for 12 hours using Heidolph Unimax 2010 shaker at the speed of 250 rpm at room temperature. Ultimately the dispersions were filtered and the final pH (pH_f_) of the solution was determined. The pHpzc was found from a plot pH_f_ vs. pH_i_. This procedure was repeated at various concentrations of 0.05 and 0.1 mol L^−1^ KNO_3_ solutions.

### 2.4 Adsorption studies

A stock solution of 1000 mg L^−1^ Cr (VI) was prepared by dissolving 2.8289 g of potassium dichromate (K_2_Cr_2_O_7_) using ultra pure water. Standard solutions for adsorption experiments were prepared by a series of dilution of the stock solution using ultra pure water. The ionic strength of the solution was attuned to 0.01, 0.05 and 0.1 mol L^−1^ using KNO_3_ as background electrolyte. The pH of the solution was adjusted in the range of 2–11 using 0.1 mol L^−1^ NaOH and HNO_3_. 5 g of VB was mixed with 100 mL of solution containing 5 mg L^−1^ Cr (VI) in a 250 mL polypropylene Erlenmeyer flask to the point of equilibrium (9 hours) without further control of pH. Control (only the test substance without adsorbent) and blank (only the adsorbent without the test substance) experiments have been carried out for each set of experiment. The flasks were tightly wrapped with polyethylene parafilm to avoid pH changes during experiments due to CO_2_ escape. The reactions were taking place at 25 ± 0.5 °C with continuous stirring at 300 rpm.

After the end of each adsorption process, it was allowed to settle for 5 minutes. Subsequently the final pH was measured. The pH changes in the experiments were observed up to a maximum value of 1. 10 mL of the supernatant sample was centrifuged and filtered through Whatman filter (pore size 2.5 μm) and the concentration of Cr (VI) was determined using UV-Vis spectrophotometer (Perkin Elmer Lambda 35, USA) by using diphenylcarbazide at maximum absorption of 540 nm [44]. Each experiment was conducted in triplicate and data represent the mean value. Before each measurement, the instruments were calibrated with standard solutions. The amount of chromium adsorbed at time t, q_t_, and the adsorbed percentage were calculated using Eqs. 1 and 2, respectively.

(1) $$q_{t\;}=\frac{\left(C_{o}-C_{t}\right)\times V}{m}$$

(2) $$A\left(\%\right)=\frac{\left(C_{o}-C_{t}\right)}{C_{o}}\times 100$$

where, $q_{t\;}\;$ the amount of chromium adsorbed per unit mass of the adsorbent (mg g^−1^), $C_{o}$ is initial concentration of chromium in contact with adsorbents (mg L^−1^), $C_{t}$ is concentration of chromium in aqueous phase at time t (mg L^−1^), V is initial volume of the aqueous phase in contact with the adsorbents (L), m is mass of the adsorbent (g) and A (%) is adsorbed amount given as percentage at time t.

### 2.5 Adsorption kinetics

The adsorption kinetic experiments for adsorption of Cr (VI) were carried in 500 mL flask containing 5 g of VB and 350 mL solutions with initial concentrations of 0.1, 1 and 5 mg L^−1^ Cr (VI) at an optimum pH and contact time between 0 to 720 min. The reactors agitation speed, temperature and volume of the sample taken and analysis were identical to the description given above in adsorption studies.

The adsorption kinetics of Cr (VI) onto the VB, experimental data was modelled by fitting pseudo-first order (3) and pseudo-second-order adsorption kinetic equations (4) [46]:

(3) $$\log\left(q_{e}-q_{t}\right)=\log\;q_{e}-\frac{k_{1}t}{2.303}$$

(4) $$\frac{t}{q_{t}}=\frac{1}{k_{2}{q_{e}}^{2}}\;+\;\frac{t}{q_{e}}$$

where $q_{e}$(mg kg^−1^) is the amounts of Cr (VI) adsorbed at equilibrium; $q_{t\;}\;$(mg kg^−1^) is the amount of Cr (VI) at time t (min), and ${\text{k}}_{1}$ (min^−1^) and ${\text{k}}_{2}\;$(kg mg^−1^min^−1^) are the rate constants of the pseudo-first-order and pseudo-second-order adsorption kinetic equations, respectively.

### 2.6 Adsorption isotherms

For adsorption isotherm studies, a series of 250 mL Erlenmeyer flasks were filled with 100 mL Cr (VI) solution of varying concentrations (0.1–5 mg L^−1^), maintained at 25 ± 0.5 °C, reaction time of 540 min and optimum pH of 2. Then 5 g VB was added into each flask. After adsorption equilibrium time the concentrations of Cr (VI) was determined using the procedure in Section 2.4. At the end of this experiment, two most commonly used isotherm adsorption models, the Langmuir and Freundlich, were used to describe the obtained equilibrium data. The linear equations of Langmuir (5) and Freundlich (6) adsorption models were expressed as follows [47].

(5) $$\frac{C_{e}}{q_{e}}=\frac{1}{b\;q_{max}}+\frac{C_{e}}{q_{max}}$$

(6) $$\log\;q_{e}=\log\;K_{f}+\frac{1}{n}\log\;C_{e}$$

where $C_{e}$ is the equilibrium concentration (mg L^−1^); $q_{e}$ is the adsorption capacity at equilibrium time (mg kg^−1^); $q_{max}$ is the maximum adsorption capacity (mg kg^−1^); b is the Langmuir constant (L mg^−1^); K_f_ is the Freundlich constant (L kg ^−1^); 1/n is the heterogeneity of the sorption sites.

## 3 Results and discussion

### 3.1 Adsorbent characterization

Instrumental techniques including FT-IR spectroscopy, Powder X-ray diffraction (XRD), scanning electron microscopy (SEM), and EDS have been employed to characterize the vesicular basalt adsorbent used in this study.

### 3.2 FT-IR analysis

**Fig. 1:**
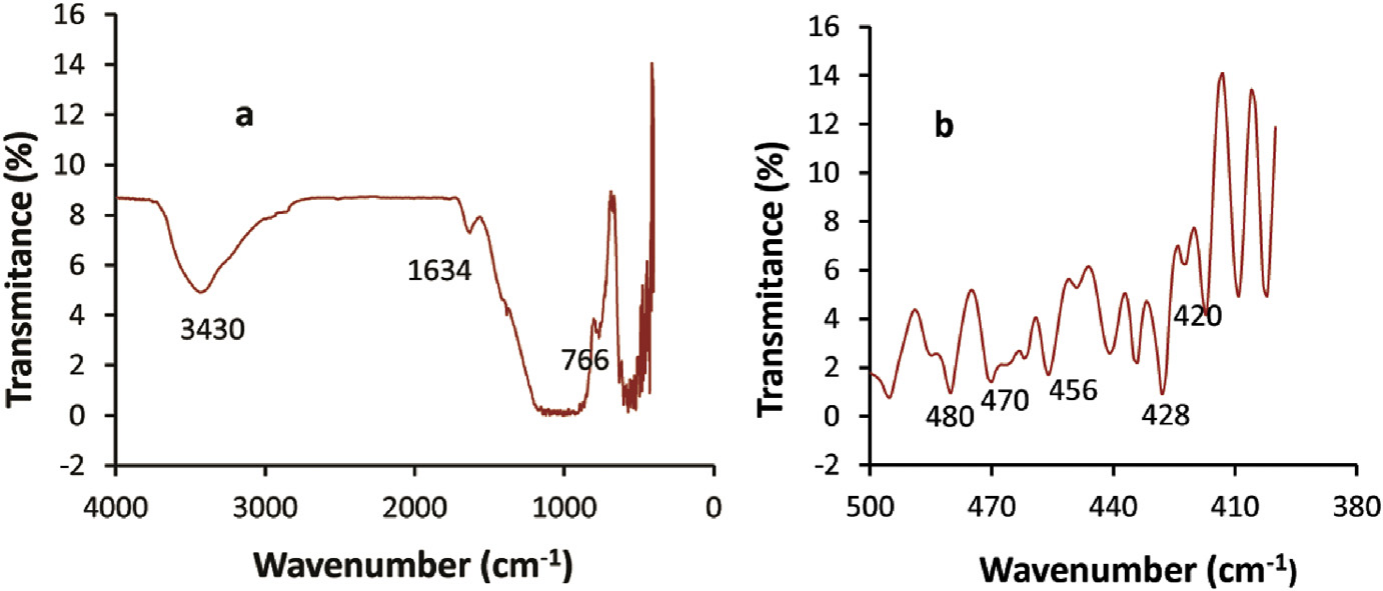
Infrared spectrum of powdered VB sample between wavenumber ranges 4000–400 cm^−1^ a); Zoom in of FT-IR spectrum of a) between wavenumber ranges 500–400 cm^−1^ b).

The FT-IR spectroscopic measurement of the VB volcanic rock is shown in Fig. 1a. The band in the region 3000–3700 cm^−1^, maximum peak at 3430 cm^−1^ was due to −OH symmetric stretching vibration and in the region 1600–1700 cm^−1^ a maximum peak at 1634 cm^−1^ was fundamental bending vibrations of H-OH [27, 48]. The spectral features displayed in the reststrahlen band region (1200–800 cm^−1^) revealed more overlap and are comparable to mineral features in the range of silica polymerization such as plagioclase, pyroxene and olivine (Fig. 1b). Such mineral composition is typical for basaltic rocks [49, 50]. In this region silicon absorption features moved to lower wave numbers owing to changes in the Si-O-Si stretching vibrations as a function of decreasing Si-O polymerization [51, 52, 53]. The FT-IR spectra showed absorption bands at 766 cm^−1^, 535 cm^−1^ and 584 cm^−1^ due to O-Si-O bending of silicates, Fe-O bending of hematite and magnetite respectively [54]. In the range of 400–600 cm^−1^ numerous vibration bands were produced at 420, 428, 456, 470 and 480 which are the characteristics of the deformation vibration of Si-O [55, 56].

### 3.3 XRD analysis

**Fig. 2:**
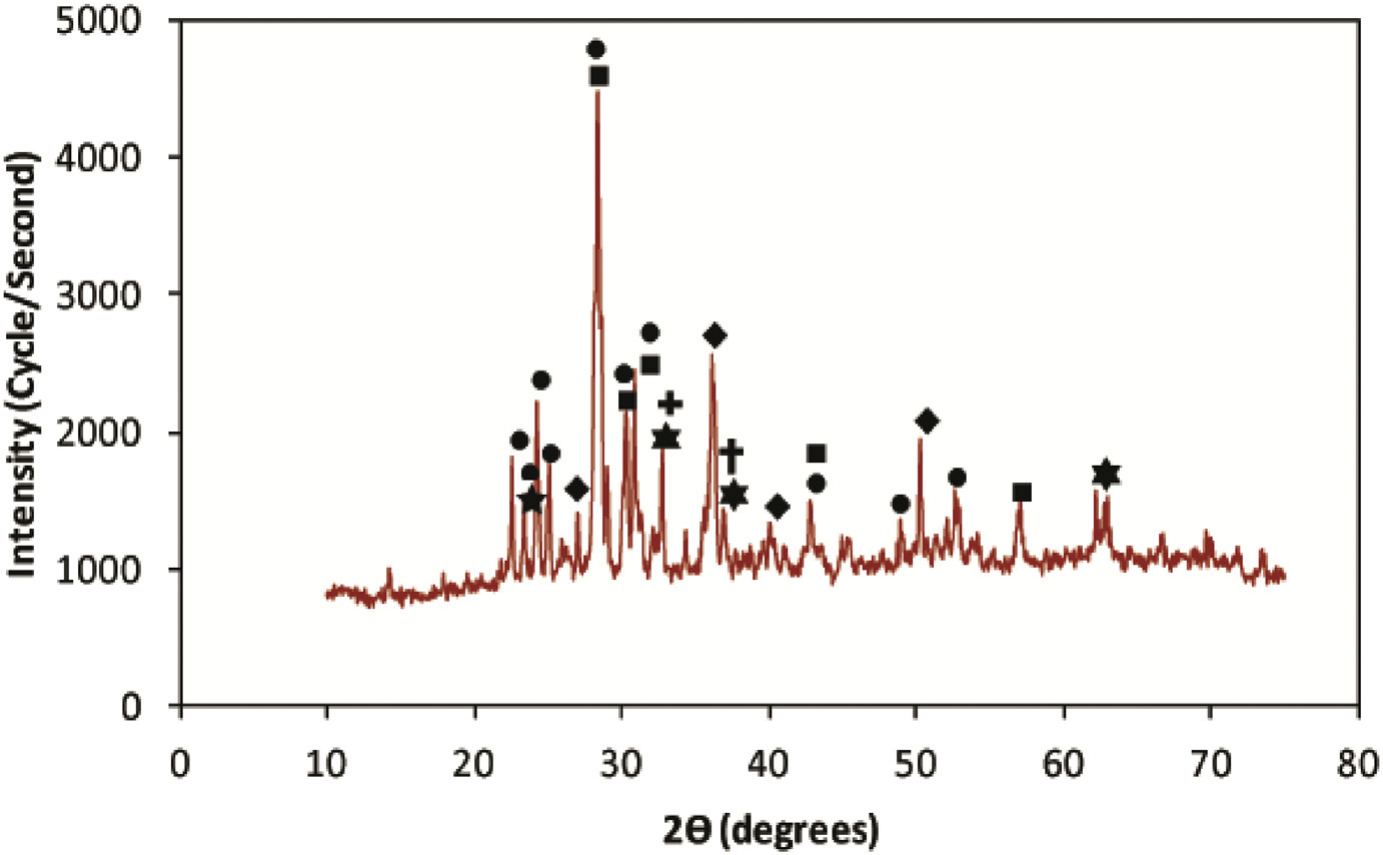
X-ray diffractogram of powdered vesicular basalt sample. Mineral assignment: () Plagioclase, () Pyroxene, () Olivine, () Quartz, () Hematite, () Goethite and () magnetite.

Identification of minerals in the VB was carried out based on the XRD patterns given by the Joint Committee for Powder Diffraction Standards (JCPDS) patterns of inorganic compounds. The X-ray diffractogram of powdered VB and its analysis is shown in Fig. 2. The diffractogram analyses of powdered VB revealed the presence of plagioclase, pyroxene (augite), quartz, olivine, goethite, hematite and magnetite, which is in agreement with other studies [57, 58, 59]. As can be seen from XRD spectrum, plagioclase is the most abundant mineral followed by Pyroxene (augite) and quartz. Moreover, the diffractogram revealed the presence of olivine, goethite, hematite and magnetite in the analyzed sample [60, 61, 62, 63]. In general, the minerals identified from the VB were in agreement with the FT-IR analyses verifying the principal minerals identified.

### 3.4 SEM/EDS analysis

**Fig. 3:**
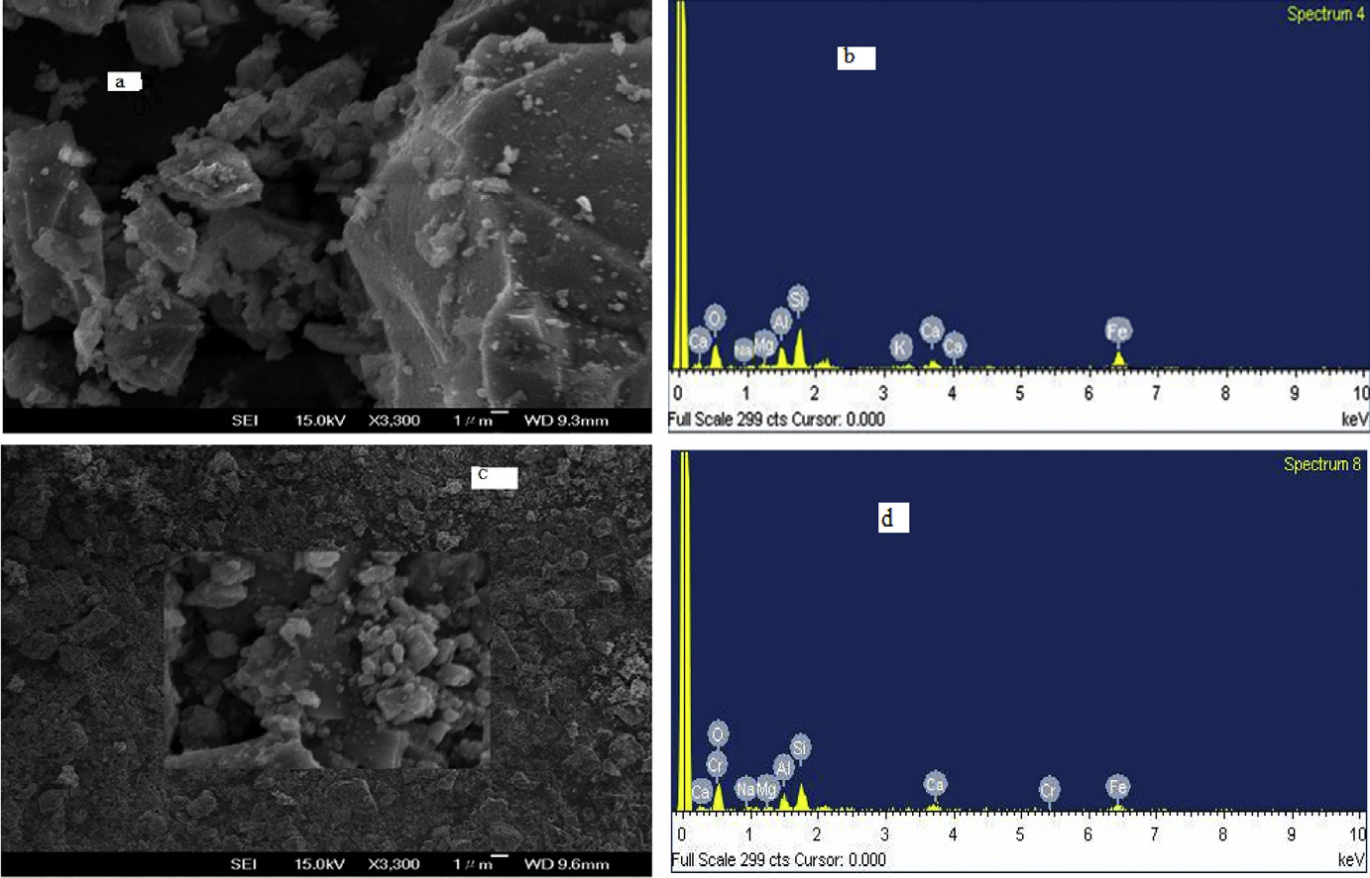
SEM image and EDS spectra of VB (a) and (b) before adsorption; SEM image and EDS spectra of VB (c) and (d) after Cr (VI) adsorption.

The SEM image and EDS analysis of the VB are provided in Fig. 3a and b below. The SEM micrographs showed homogeneous and corrugated morphology consisting of grey, light grey and white platelets of different sizes. The SEM micrographs indicate feldspars (plagioclase) with uniform grey part; light grey is the silica and white platelets are Fe-bearing aluminosilicate which is in agreement with the literature of these minerals that has been identified by direct comparison to their SEM micrographs [64, 65]. The elemental composition in Table 1a indicated that the dominant elements in the VB are oxygen (O) and silicon (Si) whose percentage compositions by weight are 48.46 and 17.37 % respectively. Other elements aluminium (Al), iron (Fe), calcium (Ca), sodium (Na), potassium (K) and magnesium (Mg) are also identified in the sample.

**Table 1:** Elemental composition of the VB before adsorption (a) and after adsorption (b) from the EDS analysis.

Element	Weight %	Atomic %
**(a)**		
O K	48.46	63.42
Mg K	0.87	0.7
Al K	9.55	6.95
Si K	17.37	14.62
Ca K	8.8	4.31
FeK	8.83	3.93
NaK	4.87	5.27
K K	1.25	0.8
Totals	100	100
**(b)**		
O K	46.41	54.63
Mg K	0.85	0.76
Al K	9.52	7.91
Si K	17.25	14.17
Ca K	7.9	5.08
FeK	8.64	6.83
NaK	4.46	7.5
Cr K	4.97	3.12
Totals	100	100

“K” represents the combined X-ray lines of K_α1_ and K_α2_ in the inner shell of an atom.

To confirm the VB in the removal of Cr (VI), SEM-EDS analysis of the exhausted adsorbent was done (Fig. 3c and d). Though it is difficult to get information about the adsorption of Cr (VI) using SEM image, from the EDS spectrum, the chromium peak is observed clearly of 4.95 % (Table 1b). The presence of this peak after adsorption process confirms that the VB has the capability to adsorb chromium (VI) from aqueous solution.

### 3.5 Effect of pH and ion strength

**Fig. 4:**
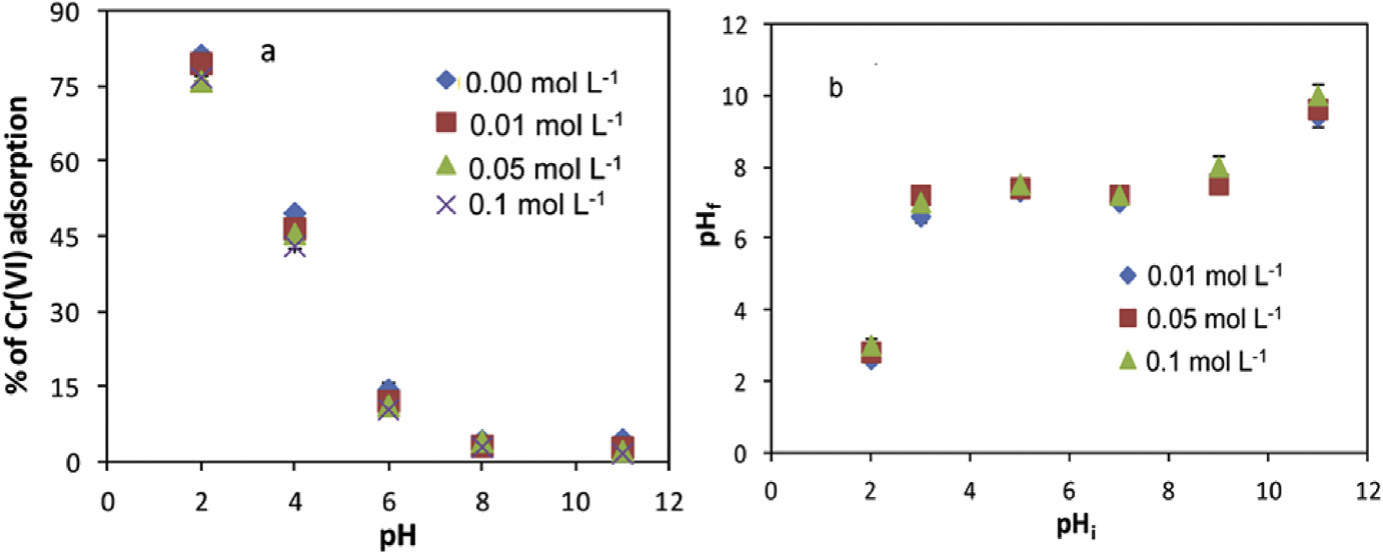
(a) Effect of pH and ionic strength for Cr (VI) adsorption onto the VB (dose 50 g L^−1^; Cr (VI) conc. 5 mg L^−1^: temperature 25 ± 0.5 °C); (b) pH_f_ Vs. pH_i_ of the VB suspension with different concentrations of KNO_3_ as a background electrolyte.

Effect of pH and ionic strength were investigated for the adsorption of Cr (VI) on vesicular basalt surface, as shown in Fig. 4a. Maximum removal of Cr (VI) was observed at acidic pH ranges with 81.19 % removal at pH 2. As the pH of the solution increased, the percentage of removal of Cr (VI) decreased and its adsorption capacity reached under 10 %. pH affected adsorption capacity of a system due to its influence on the surface properties of VB and the ionic forms of chromium in solution. In acidic condition HCrO_4_^−^ exist with higher domination, whereas the dominant species changed to CrO_4_^2−^ when pH > 6 [66]. The pHpzc of the VB at different concentrations of KNO_3_ was found to be 7.6 (Fig. 4b). After equilibration, the change in the pH_f_ was observed at lower pH_i_ (2–5) and higher pH_i_ (8–11) values. But in the pH_i_ range of 6–8, there was no significant change in the pH_f_. This implies that in this pH_i_ ranges the pHpzc is almost independent of ionic strength of KNO_3_ solution. According to Sheng et al. [67], a solid surface is positively charged at pH < pHpzc and negatively charged at pH > pHpzc. In this study at pH < 5, the surface of the VB was highly protonated with positive charges, which facilitated the adsorption of HCrO_4_^−^ species, resulting in high adsorption efficiencies [68]. When the pH > 7. 6, the surface of the VB developed less positively charged ions, which would reduce the electrostatic attractions between the VB surface and negatively charged species, lowering the adsorption efficiencies. Also, the competition of OH^−^ for the limited adsorption sites became more serious with increasing solution pH. Thus, pH 2 was taken as an optimal condition in this work. Therefore all other adsorption experiments were conducted at pH 2 to ensure for maximum removal of Cr (VI) onto the VB.

Ionic strength is another factor that affects adsorption of a system. Fig. 4a shows adsorption of Cr (VI) onto VB using different ionic strength (0, 0.01, 0.05 and 0.1 mol L^−1^) as a function of pH (2–11). The percentage of removal of Cr (VI) decreased with increasing of ionic strength in aqueous solutions. The removal of Cr (VI) decreased in the range of 1.28–4.47 % on the basis of stated ionic strength and pH ranges. This is presumed to be due to (1) competition of NO_3_^−^ with HCrO_4_^−^ or CrO_4_^2−^ with the surface of VB; (2) effect of increased ionic strength on the transformation of Cr (VI) from aqueous solutions to the VB surfaces, and (3) with increasing ionic strengths 0–0.1 mol L^−1^, the electrostatic repulsions would be reduced lowering the available active sites on VB surface [69].

### 3.6 Effect of contact time and adsorption kinetics

**Fig. 5:**
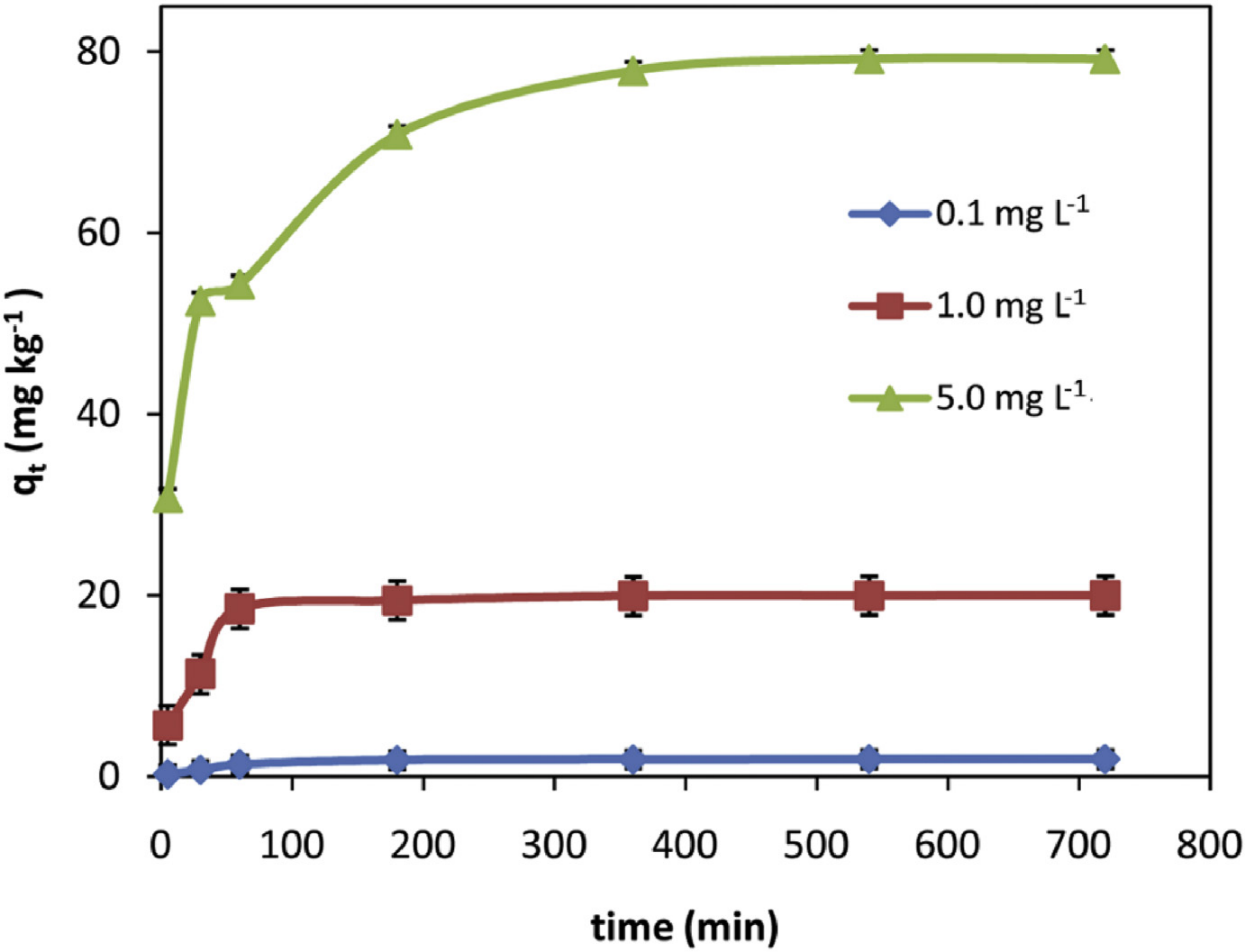
Effect of contact time on adsorption of different concentrations of Cr (VI) ions (0.1, 1.0, and 5.0 mg L^−1^) on VB. (Adsorbent dose 50 g L^−1^, solution pH 2.0 and reaction temperature 25 ± 0.5 °C).

The kinetic studies obtained for the adsorption of Cr (VI) from aqueous solutions onto VB surface are shown in Fig. 5. The time required to reach equilibrium for initial concentrations of 0.1 mg L^−1^, 1 mg L^−1^ and 5 mg L^−1^ were 180, 360 and 540 min, respectively. For lower initial concentrations (0.1 mg L^−1^), the adsorption process took place very quickly, with a maximum adsorption of 1.93 mg kg^−1^. For higher initial concentration of 5 mg L^−1^, the maximum adsorption of VB at equilibrium was 79.20 mg kg^−1^. This is in agreement with other adsorption studies of Cr (VI) from aqueous solution onto a solid surface [70, 71].

**Fig. 6:**
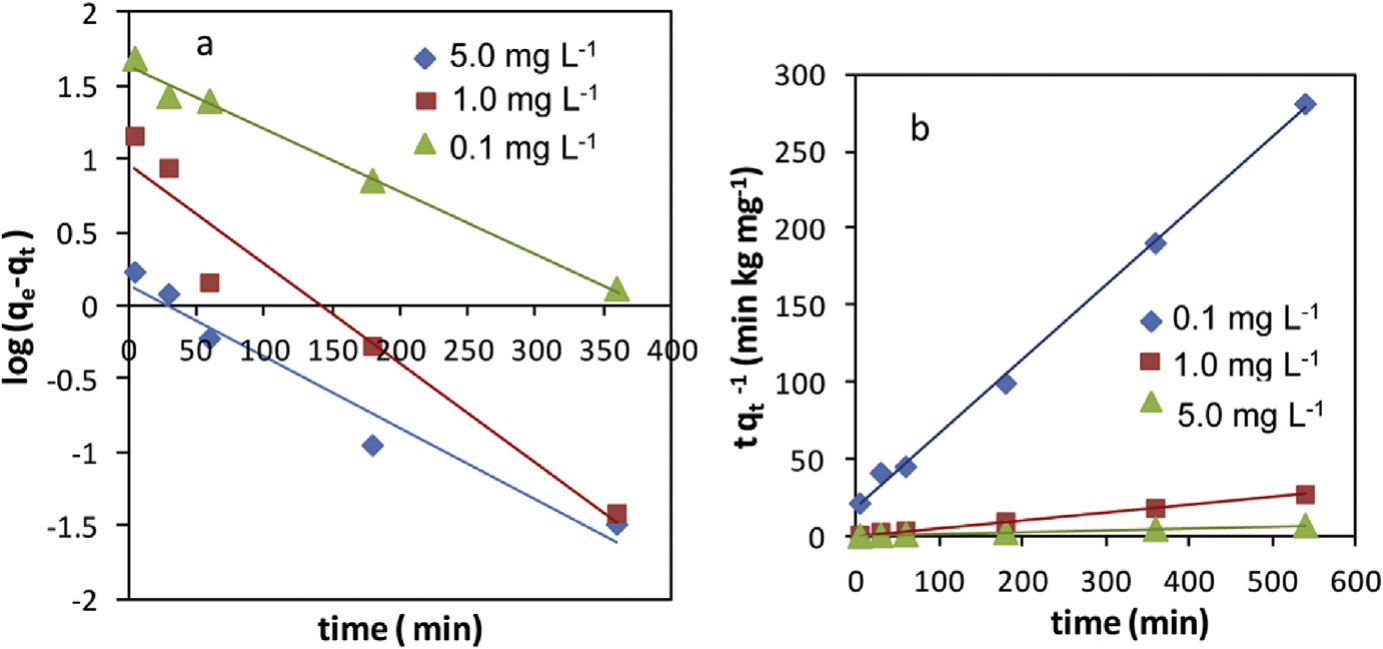
Kinetics for the adsorption of Cr (VI) onto VB: a) Pseudo-first-order; b) pseudo-second-order [(initial concentration (0.1, 1 and 5 mg L^−1^), solution pH 2, adsorbent dosage 50 mg L^−1^, temperature 25 ± 0.5 °C)].

A comparison of the results with the correlation coefficients is shown in Table 2. The correlation coefficients for the first-order kinetic model obtained at the studied concentrations (0.1, 1 and 5 mg L^−1^) were low. The $q_{e}$ values from the graph of pseudo-first-order equation (Fig. 6a) showed significant variation from experimental $q_{e}$ values. Thus this kinetic model might not be sufficient to describe the mechanism of Cr (VI)–VB interactions.

**Table 2:** Kinetic parameters for Cr (VI) adsorption onto VB.

$C_{o}\left(\mathrm{mg}L^{-1}\right)$	Pseudo-first-order			Pseudo-second-order		
	*k*_1_(*min*^−1^)	*q_e_* (*mg kg*^−1^)	*R*^2^	*k*_2_(*kg mg*^−1^*min*^−1^)	*q_e_* (*mg kg*^−1^)	*R*^2^
5	0.01	1.14	0.938	0.001	83.33	0.999
1	0.016	9.53	0.923	0.01	20.41	0.999
0.1	0.01	49.89	0.989	0.34	2.09	0.997

The plot of $\;{t\;q_{t}}^{-1}$ versus t (Fig. 6b) produces very good straight lines for different initial Cr (VI) concentrations. The correlation coefficients (R^2^) for the second-order kinetic equation were ≥0.99 for all concentrations (Table 2). The calculated $q_{e}$values also agreed well with the experimental data. These indicate that the pseudo-second- order kinetic model is suitable for describing the adsorption kinetics of Cr (VI) on VB.

### 3.7 Adsorption isotherm studies

**Fig. 7:**
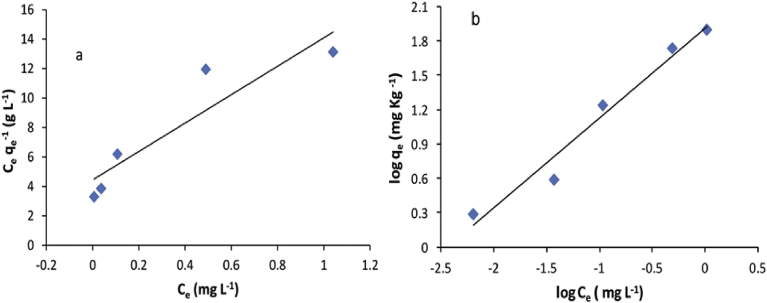
The fitting of Langmuir (a) and Freundlich (b) isotherms of Cr (VI) adsorption of onto VB volcanic rock, pH 2, dose 50 g L^−1^.

The interactive behaviour between adsorbate and adsorbent was described using Langmuir and Freundlich equilibrium adsorption models. The linear plots of Langmuir and Freundlich isotherm equations are displayed in Fig. 7a & b, respectively. Moreover, adsorption constants and correlation coefficients are shown in Table 3. The correlation coefficients (R^2^) obtained from these equations, were used as the fitting criteria to find out these models. It was found that the plots were well fitted with Freundlich isothermal adsorption models (R^2^ = 0.96). The slope (1/n) was 0.783 kg L^−1^ which is in the range of 0 and 1. This indicates that adsorption conditions were favourable and chemisorptive type of sorption was taking place rather than physical adsorption between aqueous Cr (VI) solution and VB surface [72].

**Table 3:** Langmuir and Freundlich isotherms constants for the adsorption of Cr (VI) onto VB.

Langmuir model			Freundlich Model		
q_max_ (mg kg^−1^)	b (L mg^−1^)	R^2^	K_f_ (L kg^−1^)	1/n	R^2^
104.0	2.16	0.803	6.07	0.783	0.96

The adsorption capacity of the VB for the removal of Cr (VI) was compared with other adsorbents reported in literature. The values of adsorption capacities with their experimental settings are presented in Table 4. The VB indicated good adsorption potential for Cr (VI) in aqueous solution. Based on its large availability, preparation process and general cost, it might hold superiority compared with other adsorbents and could be ideal to reduce Cr (VI) contaminated water from industries or other sources.

**Table 4:** Comparison of adsorption capacity Cr (VI) onto VB with other adsorbents.

Adsorbents	pH	Particle size (μm)	Adsorbent dosage (g L^−1^)	Metal conc. (mg L^−1^)	Adsorption capacity (mg kg^−1^)	Reference
Clay (treated)	*2.5*	*75*	20	10	200	[71]
Palygorskite clay	7	-	2	100	58480	[73]
Kaolinite	4.6	-	2	50	6100	[74]
Volcanic pumice	2	75–425	100	10	46.05	[37]
Riverbed sand	2.5	-	20	7.84	150	[75]
VB	2	90–500	50	5	79.20	This study

### 3.8 Adsorbent cost

The average cost of VB in the year 2017 was about US $14 ton^−1^ in Bahir Dar City (Ethiopia). This cost includes all expenses like transportation, electrical power for crushing, human labour etc. In Table 5 below the estimated cost of other adsorbents are compared with the naturally available VB. This price is very low and it is promising for the treatment of Cr (VI) containing wastewater for low income countries.

**Table 5:** Estimated cost of VB and other adsorbents reported in literature.

Adsorbents	Price (US $ ton^−1^)	Reference
Natural Zeolite	840.34	[76]
Fly ash	490.2	[76]
Commercial activated carbon	20000	[77]
Diatomite (for adsorbent)	92	[78]
Perlite	<1500	[79]
Commercial granular activated carbon	3300	[80]
VB	16	This study

## 4 Conclusion

In this study, VB rocks were tested for the removal of Cr (VI) from aqueous solutions. The VB was characterized using FT-IR, XRD and SEM-EDS spectroscopic techniques. The instrumental analysis indicated that plagioclase, pyroxene, silica, olivine, goethite, hematite and magnetite were the main components of VB. The influences of pH and ionic strength on the adsorption capacity of Cr (VI) onto the vesicular volcanic rock were investigated. The percentages of adsorption Cr (VI) were higher at low pH but decreased with increasing pH. At pH 2 the maximum Cr (VI) adsorbed onto the vesicular basalt volcanic rock was 81.2 %. The ionic strength reduced Cr (VI) adsorption on VB volcanic rocks to a certain extent. Kinetic studies indicated that the time required to reach adsorption equilibrium depends on the initial Cr (VI) concentrations; lower initial concentration reaches equilibrium faster than the other higher initial concentrations. The maximum adsorption capacity of Cr (VI) at equilibrium was 79.20 mg kg^−1^ at a dose of 50 g L^−1^ with initial Cr (VI) concentration of 5 mg L^−1^ at optimum pH of 2.0. It was found that the pseudo-second-order kinetic model could be used to describe the individual adsorption of Cr (VI) on VB. The adsorption isotherm data were fitted well with the Freundlich model slope (1/n) 0.78 kg L^−1^ indicating that the VB surface was heterogeneous and favourable for adsorption. Therefore, removal of Cr (VI) from polluted water by adsorption onto VB volcanic rocks as alternative low cost adsorbent appears to be feasible. Further studies have to be done using real samples from industrial effluents, along with the management options of changing the VB frequently in constructed wetlands, handing of the used VB in environmentally safe procedures and other related issues.

## Declarations

### Author contribution statement

Agegnehu Alemu: Conceived and designed the experiments; Performed the experiments; Analyzed and interpreted the data; Wrote the paper.

Brook Lemma: Conceived and designed the experiments; Analyzed and interpreted the data; Wrote the paper.

Nigus Gabiye: Conceived and designed the experiments; Analyzed and interpreted the data; Contributed reagents, materials, analysis tools or data.

Melisew Tadele Alula: Analyzed and interpreted the data; Contributed reagents, materials, analysis tools or data.

Minyahl Teferi Desta: Analyzed and interpreted the data.

### Funding statement

The work was financially supported by United States Agency for International Development (USAID) under the USAID/HED funded grant in the Africa-US Higher Education Initiative-HED 052-9740-ETH-11-01, Ethiopian Institute of Water Resources (AAU) and Biotechnology Research Institute (BDU).

### Competing interest statement

The authors declare no conflict of interest.

### Additional information

No additional information is available for this paper.

## References

[1] Cheng H., Zhou T., Li Q., Lu L., Lin C. (2014). Anthropogenic chromium emissions in China from 1990 to 2009. PLoS One.

[2] Wittbrodt P.R., Palmer C.D. (1995). Reduction of Cr (VI) in the presence of excess soil fulvic acid. Environ. Sci. Technol..

[3] Deng B., Stone A.T. (1996). Surface-catalyzed chromium (VI) reduction: the TiO_2_ - Cr (VI) - mandelic acid system. Environ. Sci. Technol..

[4] Bokare A.D., Choi W. (2011). Advanced oxidation process based on the Cr (III)/Cr (VI) redox cycle. Environ. Sci. Technol..

[5] Kotas J., Stasicka Z. (2000). Chromium occurrence in the environment and methods of its speciation. Environ. Pollut..

[6] International Agency for Research on Cancer (IARC) (1990). Chromium, nickel and welding. IARC Monogr. Eval. Carcinog. Risks Hum..

[7] Cheng L., Dixon K. (1998). Analysis of repair and mutagenesis of chromium-induced DNA damage in yeast, mammalian cells, and transgenic mice. Environ. Health Prospect..

[8] Rowbotham A.L., Levy L.S., Shuker L.K. (2000). Chromium in the environment: an evaluation of exposure of the UK general population and possible adverse health effects. J. Toxicol. Environ. Health Part B.

[9] McCarroll N., Keshava N., Chen J., Akerman G., Kligerman A., Rinde E. (2010). An evaluation of the mode of action framework for mutagenic carcinogens case study II: chromium (VI). Environ. Mol. Mutagen..

[10] Asmatulla Q.S., Shakoori A.R. (1998). Hexavalent chromium-induced congenital abnormalities in chick embryos. J. Appl. Toxicol..

[11] J. Guertin, J.A. Jacobs, C.P. Avakian, in: J. Guertin (Ed.), Chromium (VI) Handbook, Toxicity, CRC Press, Boca Raton, New York, Washington, D.C, ch.6, pp. 215–234.

[12] Agency for Toxic Substances and Disease Registry (ATSDR) (2012). A Toxicological Profile for Chromium. http://www.atsdr.cdc.gov.

[13] WHO (2011). Guidelines for Drinking-water Quality. http://www.who.int.

[14] USEPA (2009). National Primary Drinking Water Regulations.

[15] Park D., Yum Y.S., Kim J.Y., Park J.M. (2008). How to study Cr (VI) biosorption: use of fermentation waste for detoxifying Cr (VI) in aqueous solution. Chem. Eng. J..

[16] Babel S., Kurniawan T.A. (2003). Low-cost adsorbents for heavy metals uptake from contaminated water: a review. J. Hazard Mater..

[17] Petruzzelli D., Passino R., Tiravanti G. (1995). Ion exchange process for chromium removal and Recovery from tannery wastes. Ind. Eng. Chem. Res..

[18] Rengaraj S., Yeon K.H., Moon S.H. (2001). Removal of chromium from water and wastewater by ion exchange resins. J. Hazard Mater..

[19] Gao P., Chen P., Shen F., Chen G. (2005). Removal of chromium (VI) from wastewater by combined electrocoagulation-electrofloatation without a filter. Separ. Purif. Technol..

[20] Kozlowski C.A., Walkowiak W. (2002). Removal of chromium (VI) from aqueous solutions by polymer inclusion membranes. Water Res..

[21] Ge L., Wu B., Li Q., Wang Y., Yu D., Wu L., Pan J., Miao J., Xu T. (2016). Electrodialysis with nanofiltration membrane (EDNF) for high-efficiency cations fractionation. J. Membr. Sci..

[22] Deveci H., Kar Y. (2013). Adsorption of hexavalent chromium from aqueous solutions by bio-chars obtained during biomass pyrolysis. J. Ind. Eng. Chem..

[23] Babel S., Kurniawan T.A. (2004). Cr (VI) removal from synthetic wastewater using coconut shell charcoal and commercial activated carbon modified with oxidizing agents and/or chitosan. Chemosphere.

[24] Li Q., Zhai J., Zhang W., Wang M., Zhou J. (2007). Kinetic studies of adsorption of Pb (II), Cr (III) and Cu (II) from aqueous solution by sawdust and modified peanut husk. J. Hazard Mater..

[25] Owalude S.O., Tella A.C. (2016). Removal of hexavalent chromium from aqueous solutions by adsorption on modified groundnut hull. Beni-Suef Univ. J. Basic Appl. Sci..

[26] Bailey S.E., Olin T.J., Bricka R.M., Adrian D.D. (1999). A review of potentially low cost sorbents for heavy metals. Water Res..

[27] Kyziol-Komosinska J., Rosik-Dulewska C., Dzieniszewska1 A., Pająk M., Krzyzewska I. (2014). Removal of Cr (III) ions from water and wastewater by sorption onto peats and clays occurring in an overburden of lignite beds in Central Poland. Environ. Protect. Eng..

[28] Erdem E., Karapinar N., Donat R. (2004). The removal of heavy metal cations by natural zeolites. J. Colloid Interface Sci..

[29] Sprynskyy M., Buszewski B., Terzyk A.P., Namiesnik J. (2006). Study of the selection mechanism of heavy metal (Pb^2+^, Cu^2+^, Ni^2+^, and Cd^2+^) adsorption on clinoptilolite. J. Colloid Interface Sci..

[30] Sdiri A., Higashi T., Hatta T., Jamoussi F., Tase N. (2011). Evaluating the adsorptive capacity of montmorillonitic and calcareous clays on the removal of several heavy metals in aqueous systems. Chem. Eng. J..

[31] Mohan D., Singh K.P. (2002). Single and multi-component adsorption of cadmium and zinc using activated carbon derived from bagasse-an agricultural waste. Water Res..

[32] Khan T.A., Singh V.V., Ali I. (2009). Sorption of Cd(II), Pb(II) and Cr(VI) metal ions from wastewater using bottom fly ash as low cost sorbent. J. Environ. Protec. Sci..

[33] Ajmal M., Rao R.A., Anwar S., Ahmad J., Ahmad R. (2003). Adsorption studies on rice husk: removal and recovery of Cd(II) from wastewater. Bioresour. Technol..

[34] Kadirvelu K., Kavipriya M., Karthika C., Radhika M., Vennilamani N., Pattabhi S. (2003). Utilization of various agricultural wastes for activated carbon preparation and application for the removal of dyes and metal ions from aqueous solution. Bioresour. Technol..

[35] Moussavi G., Barikbin B. (2010). Biosorption of chromium (VI) from industrial wastewater on to pistachio hull waste biomass. Chem. Eng. J..

[36] Wang K., Qiu G., Cao H., Jin R. (2015). Removal of chromium (VI) from aqueous solutions using Fe_3_O_4_ magnetic polymer microspheres functionalized with amino groups. Materials.

[37] Alemayehu E., Bruhnb S.T., Lennartza B. (2011). Adsorption behaviour of Cr (VI) onto macro and micro- vesicular volcanic rocks from water. Separ. Purif. Technol..

[38] Carlson D.H., Plummer C.C., Hammersley L. (2008). Physical Geology: Earth Revealed.

[39] Mboya H.A., Kingondu C.K., Njau K.N., Mrema A.L. (2017). Measurement of pozzolanic activity index of scoria, pumice, and rice husk ash as potential supplementary cementitious materials for Portland cement. Adv. Civ. Eng..

[40] Soubrand-Colin M., Bril H., Neel C., Courtin-Nomade A., Martin F. (2005). Weathering of basaltic rocks from the French Massif Central: origin and fate of Ni, Cr, Zn and Cu. Can. Mineral..

[41] Moufti M.R., Sabtan A.A., El-Mahdy O.R., Shehata W.M. (2000). Assessment of the industrial utilization of scoria materials in central Harrat Rahat, Saudi Arabia. Eng. Geol..

[42] Benedetti M.F., Dia A., Riotte J., Chabaux F., Gerard M., Boulegue J., Fritz B., Chauvel C., Bulourde M., Deruelle B., Ildefonse P. (2003). Chemical weathering of basaltic lava flows undergoing extreme climatic conditions: the water geochemistry record. Chem. Geol..

[43] Tadesse S., Milesi J.P., Deschamps Y. (2003). Geology and mineral potential of Ethiopia: a note on geology and mineral map of Ethiopia. J. Afr. Earth Sci..

[44] American Public Health Association (APHA), American Water Works Association (AWWA), Water Environmental Federation (WEF) (2005). Standard Methods for the Examination of Water and Wastewater.

[45] Lazarevic S., Jankovic-Castvan I., Jovanovic D., Milonjic S., Janackovic D., Petrovic R. (2007). Adsorption of Pb^2+^, Cd^2+^ and Sr^2+^ ions onto natural and acid-activated sepiolites. Appl. Clay Sci..

[46] Plazinski W., Rudzinski W., Plazinska A. (2009). Theoretical models of sorption kinetics including a surface reaction mechanism: a review. Adv. Colloid Interface Sci..

[47] Betancur M., Bonelli P.R., Velasquez J.A., Cukierman A.L. (2009). Potentiality of lignin from the Kraft pulpin process for removal of trace nickel from wastewater: effect of demineralization. Bioresour. Technol..

[48] Ohlin L., Bazin P., Thibault-Starzyk F., Hedlund J., Grahn M. (2013). Adsorption of CO_2_, CH_4_, and H_2_O in Zeolite ZSM-5 studied using in situ ATR-FTIR spectroscopy. J. Phys. Chem..

[49] Kwon J.S., Yun S.T., Lee J.H., Kim S.O., Jo H.Y. (2010). Removal of divalent heavy metals (Cd, Cu, Pb, and Zn) and arsenic (III) from aqueous solutions using scoria: kinetics and equilibria of sorption. J. Hazard Mater..

[50] Morgan-Sagastume J.M., Noyola A. (2008). Evaluation of an aerobic submerged filter packed with volcanic scoria. Bioresour. Technol..

[51] Walter L.S., Salisbury J.W. (1989). Spectral characterization of igneous rocks in the 8- to 12μm region. J. Geophys. Res..

[52] Preston L.J., Izawa M.R., Banerjee N.R. (2011). Infrared spectroscopic characterization of organic matter associated with microbial bioalteration textures in basaltic glass. Astrobiology.

[53] Rajesh P., Vedhagiri S.J., Ramasamy V. (2013). FTIR characterisation of minerals in charnockite rocks of of Kalrayan Hills, India. Arch. Phys. Res..

[54] Seetha D., Velraj G. (2016). Characterization and chemometric analysis of ancient pot shards trenched from Arpakkam Tamil Nadu. India, J. Appl. Res. Technol..

[55] Maia A.A.B., Angélica R.S., Neves R.F., Pollmann H., Straub C., Saalwachter K. (2014). Use of ^29^Si and ^27^Al MAS NMR to study thermal activation of kaolinites from Brazilian Amazon Kaolin wastes. Appl. Clay Sci..

[56] Saikia N.J., Bharali D.J., Sengupta P., Bordoloi D., Goswamee R.L., Saikia P.C., Borthakur P.C. (2003). Characterization, beneficiation and utilization of kaolinite clay from Assam, India. App. Clay Sci..

[57] De Jesus Filhode F.J., David F.M., Beatriza T.G., Fabris J.D., Golart A.T., Coey M.D., Ferreira B.A., Pinto C.F. (1995). Ilmenite and magnetite of tholeiitic basalt. Clay Clay Miner..

[58] T.M. Mendes, G.P.J. Morales, P.J. Reis, Use of basaltic waste as red ceramic raw material, Ceramica, 62 (2016) 157–162.

[59] Lalla E.A., Lopez-Reyes G., Sansano A., Sanz-Arranz A., Martínez-Frías J., Medina J., Rull-Pérez F. (2016). Raman-IR vibrational and XRD characterization of ancient and modern mineralogy from volcanic eruption in Tenerife Island: implication for Mars. Geosci. Front..

[60] Parthasarathy G., Kunwar A., Srinivasan R. (2001). Occurrence of moganite-rich chalcedony in Deccan flood basalts, Killari, Maharashtra, India. Eur. J. Mineral.

[61] Costa A.C., Bigham J.M., Rhoton F.M., Traina S.J. (1999). Quantification and characterization of maghemite in soils derived from volcanic rocks in Southern Brazil. Clay Clay Miner..

[62] Hadnott B.A., Ehlmann B.L., Jolliff B.L. (2017). Mineralogy and chemistry of San Carlos high-alkali basalts: analyses of alteration with application for Mars exploration. Am. Mineral..

[63] Lalla E.A., Lopez-Reyes G., Sansano A., Sanz-Arranz A., Schmanke D., Klingelhofer G., Medina-García J., Martínez-Frías J., Rull-Pérez F. (2015). Spectroscopic analysis and XRD of terrestrial volcanic outcrops on the Tenerife Island as possible Martian analogue. Estud. Geol..

[64] Gonzalez R.M., Edwards T.E., Lorbiecke T.D., Winburn R.S., Webster J.R. (2003). Analysis of geologic Materials using rietveld quantiative X-ray diffraction. Adv. X Ray Anal..

[65] Michalski J.R., Kraft M.D., Sharp T.G., Christensen P.R. (2006). Effects of chemical weathering on infrared spectra of Columbia river basalt and spectral interpretations of Martian alteration, Earth planet. Sci. Lett..

[66] Oliveira D.Q., Goncalves M., Oliveira L.C., Guilherme L.R. (2008). Removal of as (V) and Cr (VI) from aqueous solutions using solid waste from leather industry. J. Hazard Mater..

[67] Sheng G., Li Y., Yang X., Ren X., Yang S., Hu J., Wang X. (2012). Efficient removal of arsenate by versatile magnetic graphene oxide composites. RSC Adv..

[68] Chen J., Hong X.Q., Xie Q.D., Li D.K., Zhang Q.F. (2014). Sepiolite fiber oriented-polypyrrole nanofibers for efficient chromium (VI) removal from aqueous solution. Chem. Eng..

[69] Strawn D.G., Sparks D.L. (1999). The use of XAFS to distinguish between inner- and outersphere lead adsorption complexes on montmorillonite. J. Colloid Interface Sci..

[70] Rodrigues L.A., Maschio L.J., Silva R.E., Silva M.L. (2010). Adsorption of Cr (VI) from aqueous solution by hydrous zirconium oxide. J. Hazard Mater..

[71] Khan T.A., Singh V.V. (2010). Removal of cadmium (II), lead (II), and chromium (VI) ions from aqueous solution using clay. Toxicol. Environ. Chem..

[72] Haghseresht F., Lu G. (1998). Adsorption characteristics of phenolic compounds onto coal-reject-derived adsorbents. Energy Fuels.

[73] Potgieter J.H., Potgieter-Vermaak S.S., Kalibantonga P.D. (2006). Heavy metals removal from solution by Palygorskite clay. Miner. Eng..

[74] Bhattacharyya K.G., Gupta S.S. (2006). Adsorption of chromium (VI) from water by clays. Ind. Eng. Chem. Res..

[75] Sharma Y.C., Weng C.H. (2007). Removal of chromium (VI) from water and wastewater by using riverbed sand: kinetic and equilibrium studies. J. Hazard Mater..

[76] Abdel Salam O.E., Reiad N.A., ElShafei M.M. (2011). A study of the removal characteristics of heavy metals from wastewater by low cost adsorbents. J. Adv. Res..

[77] Atun G., Hisarli G., Sheldrick W.S., Muhler M. (2003). Adsorptive removal of methylene blue from colored effluents on Fuller's Earth. J. Colloid Interface Sci..

[78] Crangle R.D. (August, 2014). Diatomite Minerals Yearbook (2012).

[79] Mathialagan T., Viraraghavan T. (2002). Adsorption of cadmium from aqueous solutions by perlite. J. Hazard Mater..

[80] Toles C.A., Marshall W.E., Wartelle L.H., McAloon A. (2000). Steam or carbon dioxide-activated carbons from almond shells: physical, chemical and adsorptive properties and estimated cost of production. Bioresour. Technol..

