# Corrigendum to “Removal of chromium (VI) from aqueous solution using vesicular basalt: A potential low cost wastewater treatment system” [Heliyon 4(7) (July 2018), Article e00682]

**DOI:** 10.1016/j.heliyon.2018.e00712

**Published:** 2018-07-31

**Authors:** Agegnehu Alemu, Brook Lemma, Nigus Gabbiye, Melisew Tadele Alula, Minyahl Teferi Desta

**Affiliations:** a Ethiopian Institute of Water Resources, Addis Ababa University, P.O Box 1176, Addis Ababa, Ethiopia; b College of Science, Bahir Dar University, P.O Box 79, Bahir Dar, Ethiopia; c College of Natural and Computational Science, Addis Ababa University, P.O Box 1176, Addis Ababa, Ethiopia; d Faculty of Chemical and Food Engineering, Bahir Dar University, P.O. Box 26, Bahir Dar, Ethiopia; e Botswana International University of Science and Technology, Private Bag 16, Botswana; f School of Earth Science, Bahir Dar University, P.O Box 79, Bahir Dar, Ethiopia

In the original published version of this article, an error was present in the author list. The 4th author was listed incorrectly as “Melisew Tadele” instead of the correct name “Melisew Tadele Alula” and the 5th author was listed incorrectly as “Minyahil Teferi” instead of the correct name “Minyahl Teferi Desta”. The authors apologize for the mistake. Both the HTML and PDF versions of the article have been updated to correct the error.

